# The complete chloroplast genome of *Streblus indicus*

**DOI:** 10.1080/23802359.2019.1660246

**Published:** 2019-09-02

**Authors:** Dejun Yang, Qiong Qiu, Linhong Xu, Yumei Xu, Yi Wang

**Affiliations:** Institute of Tropical Forestry, Yunnan Academy of Forestry, Puwen, People's Republic of China

**Keywords:** *Streblus indicus*, chloroplast, Illumina sequencing, phylogenetic analysis

## Abstract

The first complete chloroplast genome sequences of *Streblus indicus* were reported in this study. The cpDNA of *S. indicus* is 159,853 bp in length, contains a large single copy region (LSC) of 88,950 bp and a small single copy region (SSC) of 19,313 bp, which were separated by a pair of inverted repeat (IR) regions of 25,795 bp. The genome contains 129 genes, including 84 protein-coding genes, eight ribosomal RNA genes, and 37 transfer RNA genes. The overall GC content of the whole genome is 36.1%. Phylogenetic analysis of 14 chloroplast genomes within the family Moraceae shows that *S. indicus* clustered in a unique clade.

*Streblus indicus* belongs to the genus *Streblus* in Moraceae family and is a wild perennial arbor, which is widespread in South Asia, particularly in southern China (Zhang et al. 1998). *Streblus indicus* is a traditional medicine in Chinese, which is often used for hemostasis and the treatments of inflammation and various rheumatoid diseases (Zhao et al. 1999; He et al. 2017). He et al. (2016) reported that the bark extract of *S. indicus* showed inhibitory activity against A549 and MCF-7 tumor cells. It is the ingredients of Chinese patent medicine Yunnan red medicine capsule (Liu et al. 2018). *Streblus indicus* is also important urban afforestation tree species in southern China (Yang et al. 2011). However, there has been no genomic studies on *S. indicus*.

Herein, we reported and characterized the complete *S. indicus* plastid genome (MN065161). One *S. indicus* individual (specimen number: 201806011) was collected from Puwen, Yunnan Province of China (22°49′43″ N, 101°9′27″ E). The specimen is stored at Yunnan Academy of Forestry Herbarium, Kunming, China, and the accession number is YAFH0012756. DNA was extracted from its fresh leaves using DNA Plantzol Reagent (Invitrogen, Carlsbad, CA, USA).

Paired-end reads were sequenced by using Illumina HiSeq system (Illumina, San Diego, CA). In total, about 27.1 million high-quality clean reads were generated with adaptors trimmed. Aligning, assembly, and annotation were conducted by CLC de novo assembler (CLC Bio, Aarhus, Denmark), BLAST, GeSeq (Tillich et al. 2017), and GENEIOUS v 11.0.5 (Biomatters Ltd, Auckland, New Zealand). To confirm the phylogenetic position of *S. indicus*, other 13 species of family Moraceae from NCBI were aligned using MAFFT v.7 (Katoh and Standley 2013) and maximum-likelihood (ML) bootstrap analysis was conducted using RAxML (Stamatakis 2006); bootstrap probability values were calculated from 1000 replicates. *Cecropia pachystachya* (MF953831) and *Debregeasia orientalis* (MH196364) were served as the out-group.

The complete *S. indicus* plastid genome is a circular DNA molecule with the length of 159,853 bp, with large single copy (LSC: 88,950 bp), small single copy (SSC: 19,313 bp), and two inverted repeats (IRa and IRb: 25,795 bp each). The overall GC content of the whole genome is 36.1%, and the corresponding values of the LSC, SSC, and IR regions are 33.7%, 29.3%, and 42.7%, respectively. The genome contains 129 genes, including 84 protein-coding genes, eight ribosomal RNA genes, and 37 transfer RNA genes. Phylogenetic analysis showed that *S. indicus* clustered in a unique clade, which indicated the phylogenesis classification of *S. indicus* (Figure 1). The determination of the complete plastid genome sequences provided new molecular data to illuminate the Moraceae evolution.

**Figure 1.**
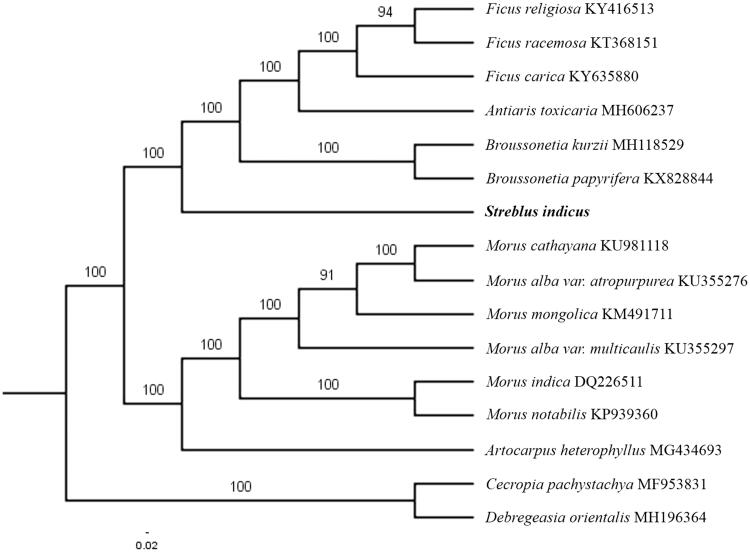
The maximum-likelihood tree based on the 14 chloroplast genomes of family Moraceae. The bootstrap value based on 1000 replicates is shown on each node.
